# Hydralazine augmented ultrasound hyperthermia for the treatment of hepatocellular carcinoma

**DOI:** 10.1038/s41598-021-94323-0

**Published:** 2021-07-30

**Authors:** Mrigendra B. Karmacharya, Laith R. Sultan, Stephen J. Hunt, Chandra M. Sehgal

**Affiliations:** 1 Ultrasound Research Laboratory, Department of Radiology, Perelman School of Medicine, University of Pennsylvania, 3620 Hamilton Walk, Philadelphia, PA 19104 USA; 2 Penn Image-Guided Interventions Lab, Department of Radiology, Perelman School of Medicine, University of Pennsylvania, 421 Curie Blvd, 646 BRB II/III, Philadelphia, PA 19104 USA

**Keywords:** Cancer therapy, Cancer imaging

## Abstract

This study investigates the use of hydralazine to enhance ultrasound hyperthermia for the treatment of hepatocellular carcinoma (HCC) by minimizing flow-mediated heat loss from the tumor. Murine HCC tumors were treated with a continuous mode ultrasound with or without an intravenous administration of hydralazine (5 mg/kg). Tumor blood flow and blood vessels were evaluated by contrast-enhanced ultrasound (CEUS) imaging and histology, respectively. Hydralazine markedly enhanced ultrasound hyperthermia through the disruption of tumor blood flow in HCC. Ultrasound treatment with hydralazine significantly reduced peak enhancement (PE), perfusion index (PI), and area under the curve (AUC) of the CEUS time-intensity curves by 91.9 ± 0.9%, 95.7 ± 0.7%, and 96.6 ± 0.5%, compared to 71.4 ± 1.9%, 84.7 ± 1.1%, and 85.6 ± 0.7% respectively without hydralazine. Tumor temperature measurements showed that the cumulative thermal dose delivered by ultrasound treatment with hydralazine (170.8 ± 11.8 min) was significantly higher than that without hydralazine (137.7 ± 10.7 min). Histological assessment of the ultrasound-treated tumors showed that hydralazine injection formed larger hemorrhagic pools and increased tumor vessel dilation consistent with CEUS observations illustrating the augmentation of hyperthermic effects by hydralazine. In conclusion, we demonstrated that ultrasound hyperthermia can be enhanced significantly by hydralazine in murine HCC tumors by modulating tumor blood flow. Future studies demonstrating the safety of the combined use of ultrasound and hydralazine would enable the clinical translation of the proposed technique.

## Introduction

Hepatocellular carcinoma (HCC), one of the most prevalent primary liver cancers and a leading cause of cancer-related death worldwide, is a highly vascular solid tumor and its development critically depends on angiogenesis^1–3^. Several treatment options for HCC are available depending on its stage and the underlying hepatic pathophysiology. For early-stage HCC patients, surgical resection and liver transplantation are principal treatments^4^. However, due to its asymptomatic nature in the early stages of the tumor, the majority of the HCC cases are detected in the advanced stages of the disease^5,6^. For intermediate and advanced-stage patients, transarterial chemoembolization (TACE), radioembolization, and systemic therapies are most commonly performed. The management of advanced HCC continues to be challenging, and the prognosis in the advanced stages remains poor. Newer treatment methods are needed, and a broad range of loco-regional and systemic options with ablative, radiation, and pharmaceuticals are under development to enhance HCC treatment^4–7^.

We have previously demonstrated the capability of ultrasound for the treatment of HCC and other cancers^8–12^. Cellular and molecular vibrations during longitudinal ultrasound wave oscillations cause tumor tissues to absorb acoustic energy and induce localized hyperthermia. Hyperthermia has long been used as an adjunctive radio- and chemo-sensitizing therapeutic agent for HCC treatment^13–15^. Hyperthermia has shown to enhance radiation response in HCC by increasing radiation-induced cytotoxicity and inhibiting DNA damage repair mechanisms^16^. Under the normal course of hyperthermia, the presence of blood vessels in the targeted tissue region buffers the adjacent cells from the intended tissue damage by allowing thermal energy to dissipate via blood flow^17,18^. While this phenomenon helps to protect the untargeted neighboring tissue-heating, it also reduces tumor hyperthermia, especially in highly vascular tumors like HCC. Therefore, for an ideal thermal therapy for HCC, there is a need for minimizing the unintended non-tissue-specific hyperthermia and simultaneously maximizing the loco-regional tumor-specific heating. Ideally, this could be achieved by modulating tumor blood flow in HCC tumors.

In this study, we propose a new approach of ultrasound-induced hyperthermia for HCC therapy by reducing the flow-mediated heat loss in the tumors following the intravenous administration of hydralazine. Hydralazine, a commonly used oral antihypertensive drug and a direct-acting peripheral arterial vasodilator^19^, exerts its vasodilatory effects by modifying the contractile state of the arterial vascular smooth muscle cells altering intracellular Ca^2+^ release^20^. Several studies have demonstrated hydralazine to enhance hyperthermia in various tumor models^21–26^. Hydralazine has been shown to markedly increase tumoricidal effects of heat therapy in mice Ehrlich carcinoma where the anti-tumor therapeutic effects were related to the tumor blood flow^21^. Hydralazine has been shown to enhance selective tumor heating and augment local heat therapy of the transmissible venereal tumor implants in dogs^22^. Additionally, hydralazine enhanced thermal damage in murine SCC-VII tumors by modifying blood flow and oxygen tension^23^, local heat damage in C3H mammary carcinoma in vivo^24^, and tumor susceptibility of hyperthermochemotherapy in B16 melanoma tumors in mice^26^.

Classically, hydralazine has been reported to reduce intra-tumor blood flow from the ‘steal’ effect^24,25^. It induces peripheral vasodilation in normal tissues, thus ‘stealing’ the blood away by the tumor^27^. The highly vascular and aberrant nature of HCC tumor blood vessels provides a unique paradigm for vasomodulation of tumor blood flow and is yet to be exploited. We hypothesized that the administration of hydralazine reduces intra-tumor blood flow and subsequently enhances the efficacy of ultrasound hyperthermia in HCC. This murine study investigates the effects of hydralazine on the efficacy of ultrasound hyperthermia by real-time image-guided contrast-enhanced ultrasound (CEUS) imaging and histological studies.

## Results

### Hydralazine significantly decreased blood flow in HCC

**Figure 1:**
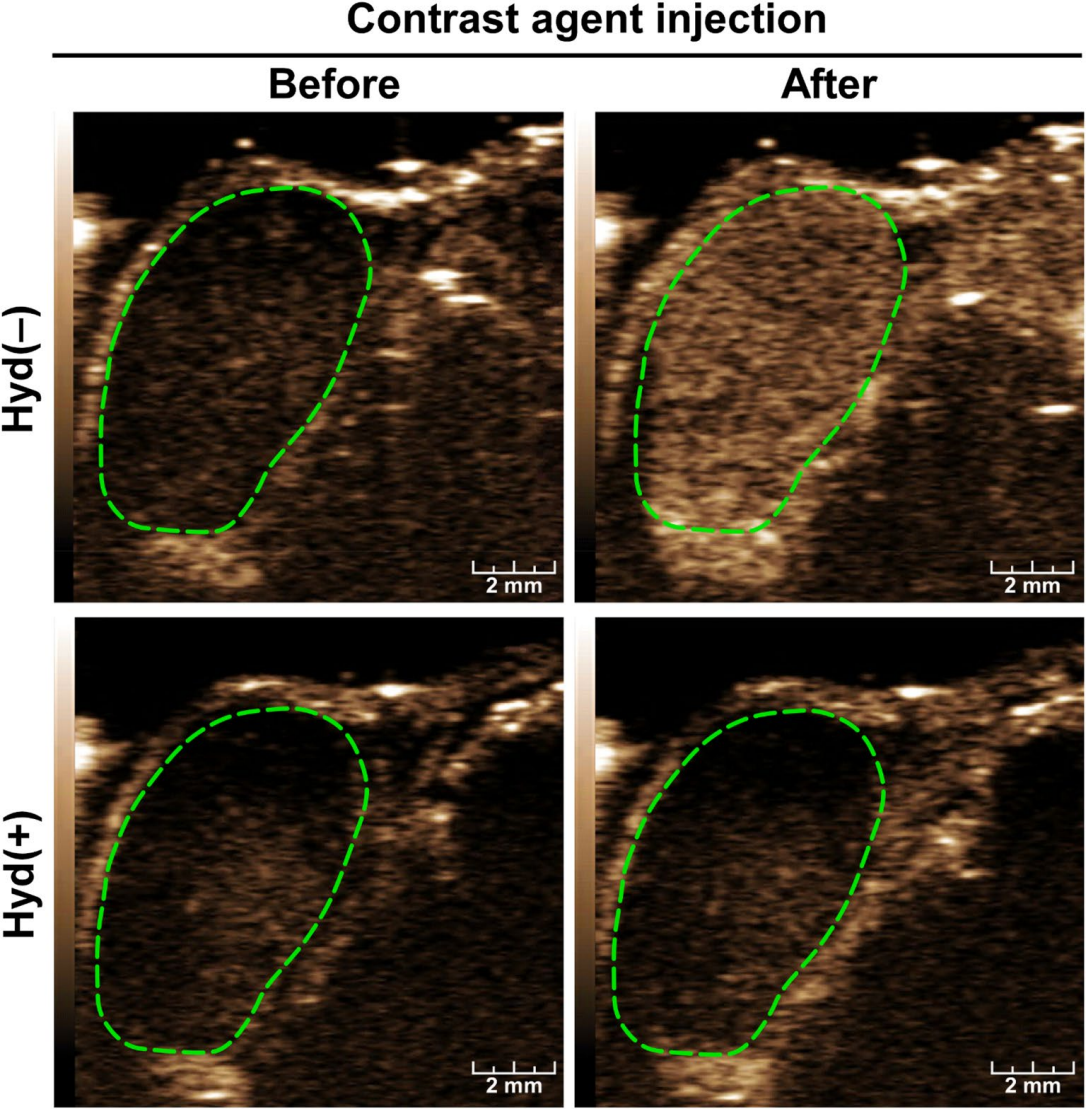
Effects of hydralazine alone on HCC blood flow. Non-linear contrast-enhanced (NLC) images of the tumor were acquired before (left panel) and after (right panel) intravenous injections of the contrast agent with [Hyd(+)] or without [Hyd(−)] administration of 5 mg/kg hydralazine; the margins of the tumor defined by the green dotted lines were outlined on the grayscale images (not shown) and copied to the NLC image acquired simultaneously. The CEUS images demonstrated that before hydralazine administration the tumor and the surrounding tissues were highly vascular, represented by the inflow of contrast agent (panels in the top row). The inflow of contrast agent through the tumor was substantially reduced following hydralazine treatment (panels in the bottom row). Scale bar = 2 mm.

**Figure 2:**
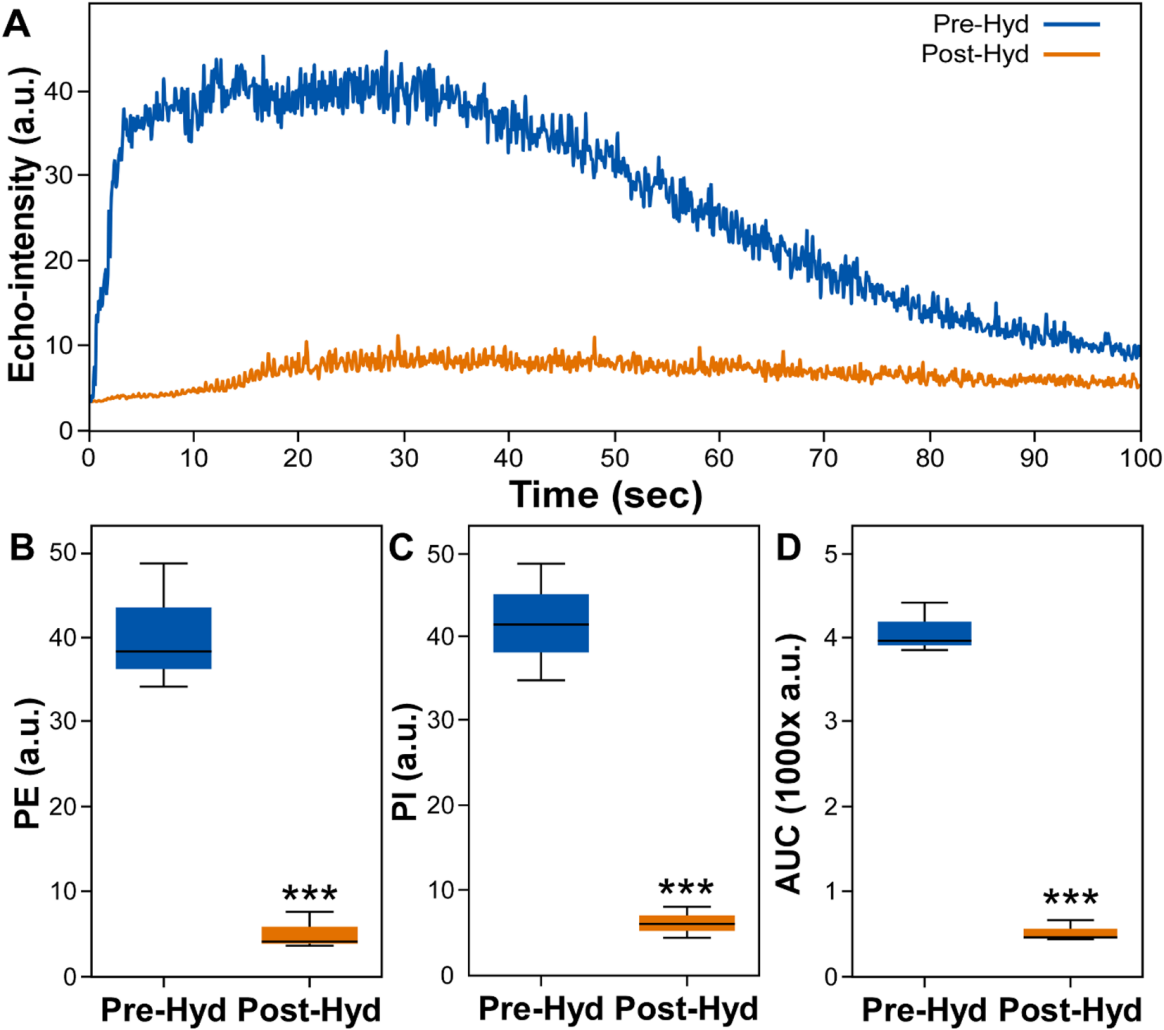
Effects of hydralazine alone on HCC blood flow as shown by quantitative analyses of the CEUS images. The time-intensity curves (**A**) displaying echo-intensity (arbitrary units, a.u.), plotted over time before (Pre-Hyd) and after (Post-Hyd) hydralazine treatment. Note that hydralazine caused a substantial decrease in HCC echogenicity. Boxplots (**B**–**D**) illustrate the five-number summary, the minimum, first quartile, median, third quartile, and maximum value of peak enhancement (PE), perfusion index (PI), and area under the curve (AUC) respectively. All data points for PE, PI, and AUC for Pre-Hyd and Post-Hyd groups were normally distributed by the Shapiro–Wilk test (*p*-value > 0.05). Following hydralazine treatment, there were significant reductions in the CEUS vascular imaging parameters (***p ≤ 0.001).

Intravenous administration of hydralazine at the concentration of 5 mg/kg significantly reduced CEUS signals in mouse HCC tumors. In the absence of hydralazine [Hyd(−)], CEUS images showed that the tumors were highly and uniformly vascular (top panels, Fig. 1). Following hydralazine [Hyd(+)], each tumor showed a dramatic reduction in contrast enhancement (lower panels, Fig. 1). The enhancement of the images by the contrast agent over time represented by the time-intensity curves were markedly attenuated following hydralazine injection (Fig. 2A). After hydralazine injection, each of the vascular imaging parameters was significantly reduced (*p* < 0.001) (Fig. 2B–D)—peak enhancement (PE) pre-treatment = 43.4 ± 5.2, post-treatment = 5.8 ± 1.8, reduction = 86.9 ± 2.5%; perfusion index (PI) pre-treatment = 44.6 ± 3.5, post-treatment = 6.3 ± 1.8, reduction = 85.8 ± 2.7%; area under the curve (AUC) pre-treatment = 3871.1 (± 943.6), post-treatment = 112.4 ± 54.9, reduction = 86.1 ± 2.9%. There was no significant change (*p* > 0.9) in these parameters in the sham group where saline was injected instead of hydralazine—PE pre-sham = 35.9 ± 4.3, post-sham = 36.1 ± 5.1; PI pre-sham = 33.9 ± 2.8, post-sham = 35.3 ± 3.7; AUC pre-sham = 3264.9 ± 270.8, post-sham = 3424.1 ± 168.1. Notably, in the non-tumor tissue adjacent to the tumors the time-intensity curves, and PE, PI, and AUC values did not decrease before and after hydralazine injections (Supplementary Fig. S1).

### Hydralazine enhanced antivascular effects of ultrasound in HCC

**Figure 3:**
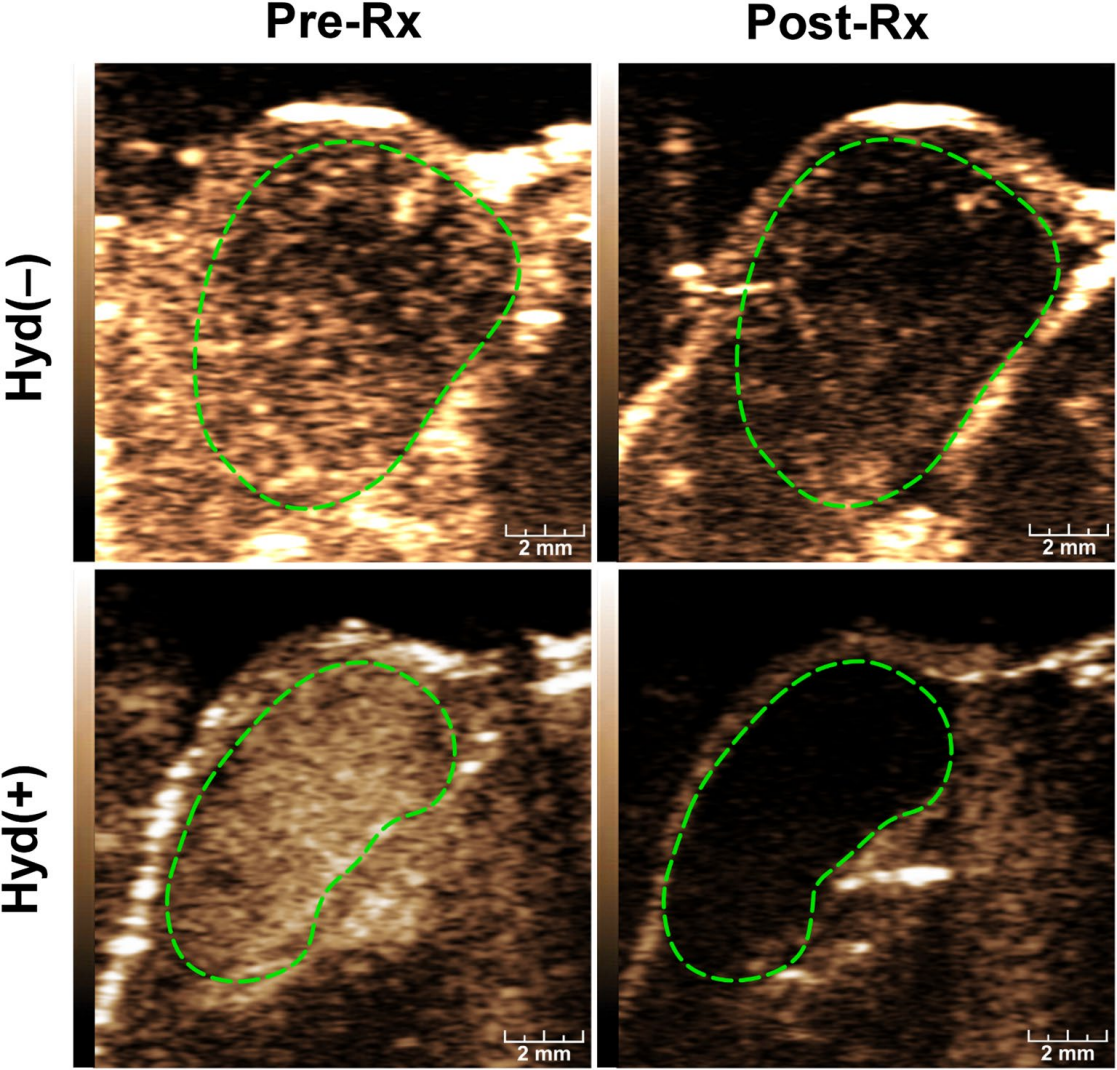
Effects of combined ultrasound and hydralazine treatments on HCC blood flow. Non-linear contrast-enhanced (NLC) images were acquired prior to (Pre-Rx) and following (Post-Rx) ultrasound treatment with [Hyd(+)] or without [Hyd(−)] administration of 5 mg/kg hydralazine; the margins of the tumor were outlined in dotted lines on the grayscale images (not shown) were copied to the NLC image acquired simultaneously. The CEUS images demonstrate that the addition of hydralazine to ultrasound therapy resulted in a further substantial loss in tumor vascularity. Scale bar = 2 mm.

**Figure 4:**
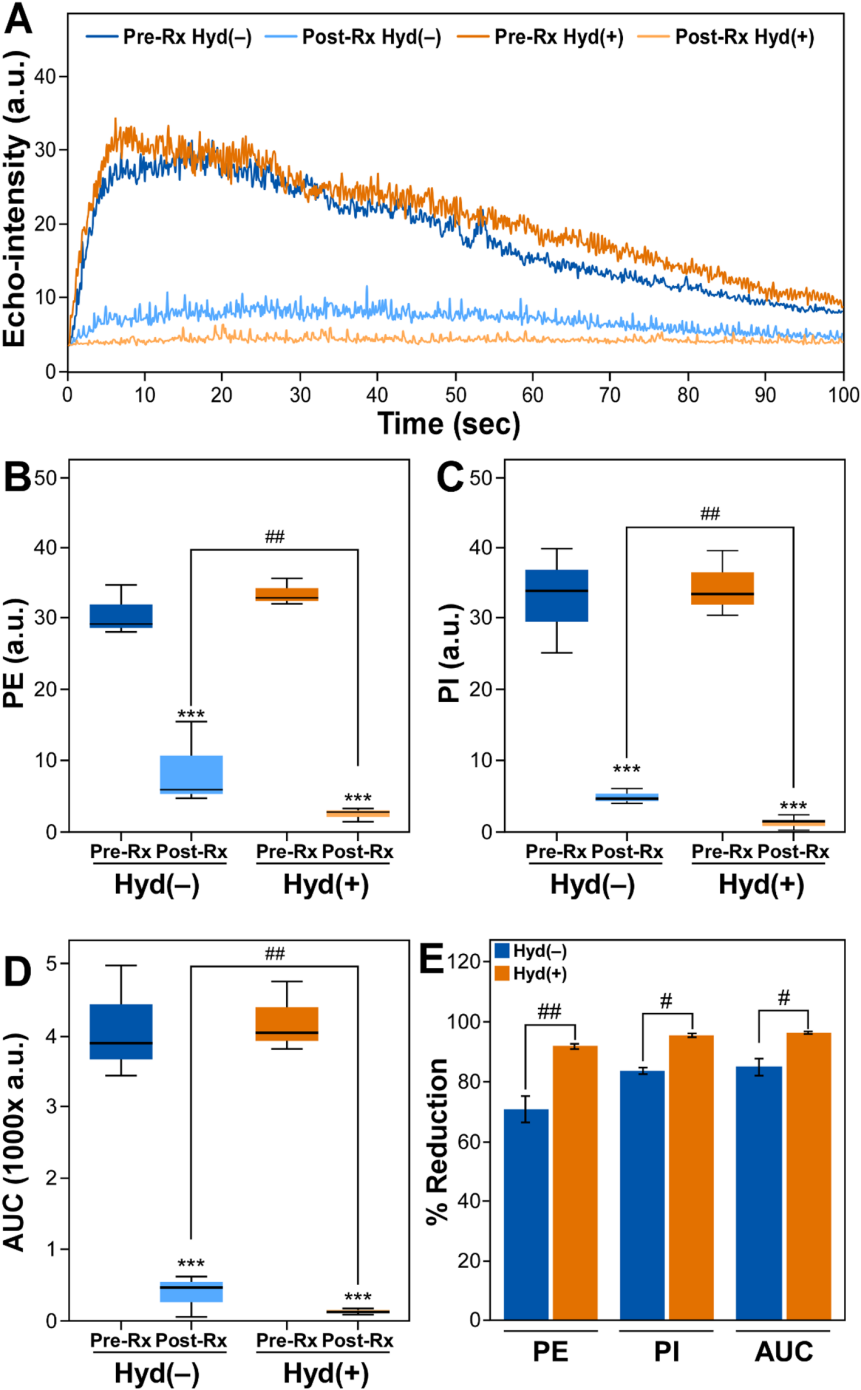
Effects of combined ultrasound and hydralazine treatments on HCC blood flow as measured by quantitative analyses of the CEUS images. Time**-**intensity curves (**A**) displaying echo-intensity (arbitrary units, a.u.) plotted over time prior to and following ultrasound therapy with and without the administration of hydralazine; Pre-Rx Hyd(−) = pre-ultrasound therapy without hydralazine; Post-Rx Hyd(−) = post-ultrasound therapy without hydralazine; Pre-Rx Hyd(+) = pre-ultrasound therapy with hydralazine; Post-Rx Hyd(+) = post-ultrasound therapy with hydralazine. Boxplots (**B**–**D**) illustrate the five-number summary, the minimum, first quartile, median, third quartile, and maximum value of peak enhancement (PE), perfusion index (PI), and area under the curve (AUC) respectively. All data points for PE, PI, and AUC for all experimental groups were normally distributed by the Shapiro–Wilk test (*p*-value > 0.05). Note that PE, PI and AUC were significantly decreased after ultrasound therapy in both Hyd(−) and Hyd(+) groups. The difference in the average values (± SEM) of PE, PI, and AUC between Pre-Rx vs. Post-Rx was statistically significant (***p ≤ 0.001). The combined hydralazine and ultrasound therapy caused the larger decrease in HCC echogenicity which was related to a reduction in tumor vascularity. Bar graphs (**E**) demonstrate the percentage reduction in PE, PI, and AUC after ultrasound therapy between Hyd(−) and Hyd(+) groups (^#^*p* ≤ 0.05, ^##^*p* ≤ 0.01). Note that the reduction in the CEUS vascular imaging parameters in the combined therapy is significantly lower compared to the ultrasound-alone treatment.

Treatment with ultrasound alone decreased the contrast enhancement of HCC (top panels, Fig. 3) and administration of hydralazine further decreased the CEUS signal (lower panels, Fig. 3). Time-intensity curves of the contrast-enhanced images of ultrasound-treated tumors showed a greater change in the presence of hydralazine (Fig. 4A). The combination of hydralazine and ultrasound significantly reduced (*p* < 0.001) the CEUS parameters (Fig. 4B–E)—PE pre-treatment = 34.2 ± 0.7, post-treatment = 2.8 ± 0.4, reduction = 91.93 ± 0.95%; PI pre-treatment = 36.1 ± 1.7, post-treatment = 1.6 ± 0.3, reduction = 95.68 ± 0.7% AUC pre-treatment = 4413.5 ± 177.9, post treatment = 148.6 ± 15.9, reduction = 96.6 ± 0.5%. When ultrasound was administered without hydralazine, there were also reductions (*p* < 0.001) in the CEUS parameters—PE pre-treatment = 32.1 ± 1.3, post-treatment = 11.1 ± 2.17, reduction = 71.4 ± 1.9%; PI pre-treatment = 35.2 ± 2.7, post-treatment = 5.3 ± 0.4, reduction = 84.7 ± 1.1%; AUC pre-treatment = 4436.3 ± 286.3, post-treatment = 476.5 ± 105.4, reduction = 85.6 ± 0.7%. These later reductions in tumor vascularity were not, however, as large as those observed when hydralazine was combined with ultrasound; the difference between the Hyd(−) and Hyd(+) groups was statistically significant for each of the imaging parameters (Fig. 4E): PE (*p* < 0.01); PI (*p* < 0.03); and AUC (*p* < 0.01). In the sham-treated group, there was no significant change (*p* > 0.9) in the imaging parameters—PE pre-treatment = 35.9 ± 4.3, post treatment = 34.2 ± 5.5; PI pre-treatment = 33.9 ± 2.8, post-treatment = 35.4 ± 4.7; AUC pre-treatment = 3264.9 ± 270.8, post-treatment = 3310.9 ± 315.5.

### Hydralazine augmented thermal dose of ultrasound hyperthermia on HCC tumors

**Figure 5:**
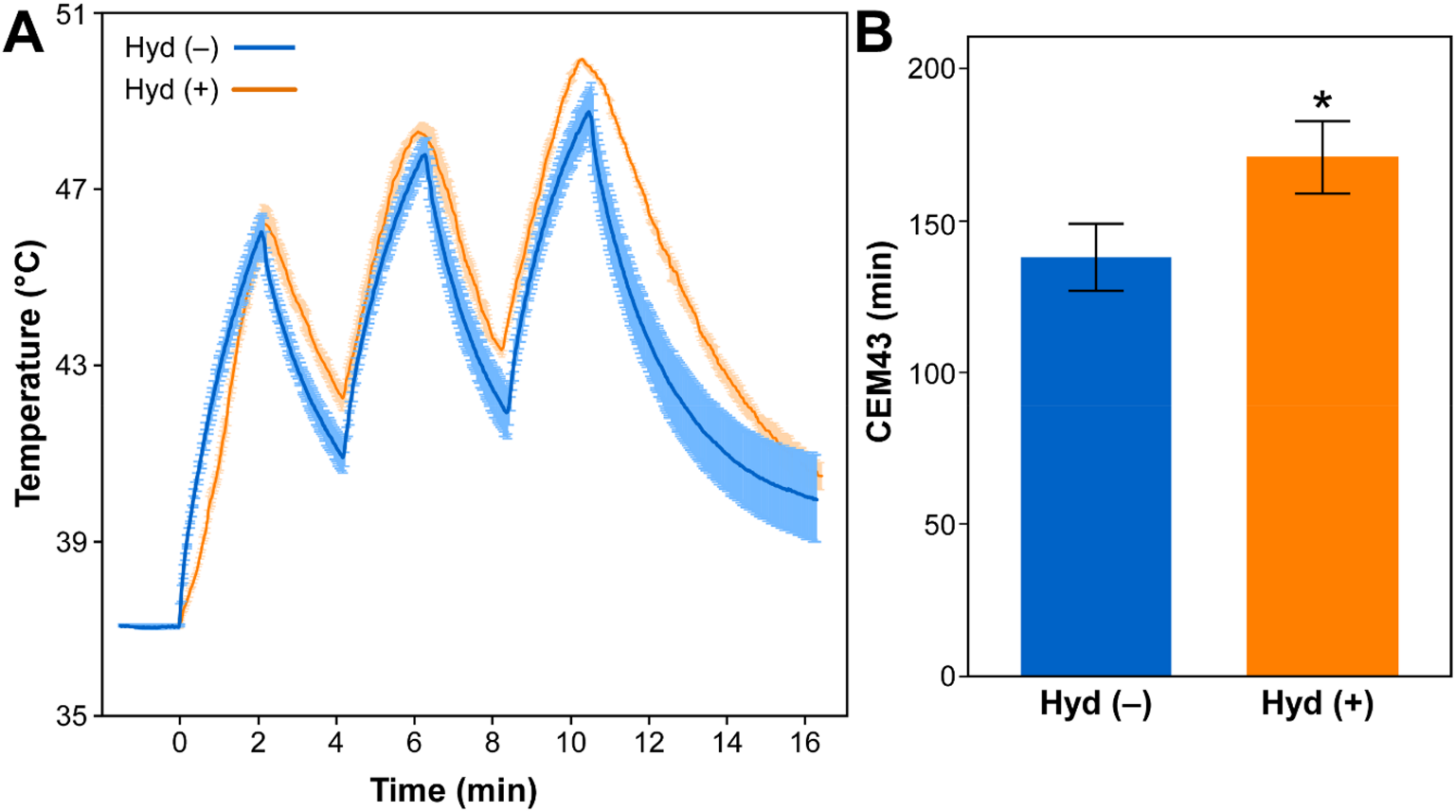
Tumor temperature and thermal dose (CEM43) measurement. (**A**) Graphs showing the average tumor temperatures (± SEM, colored bands) in live mice during ultrasound treatment with [orange, Hyd(+)] or without [blue, Hyd(−)] hydralazine. (**B**) Graph showing thermal dose (CEM43) calculated from the tumor temperature data for the ultrasound treatment groups with [orange, Hyd(+)] or without [blue, Hyd(−)] hydralazine. The difference in CEM43 values between Hyd(−) and Hyd(+) groups is statistically significant (*p* < 0.05).

Tumor temperature measured in vivo in mice tumors showed that treatment with ultrasound alone (three 2–2 min on–off cycles) delivered substantial thermal dose—expressed as the cumulative equivalent minutes at 43 °C (CEM43)—to the tumors. Tumor temperature during ultrasound treatment in the presence of hydralazine [Hyd(+)] was significantly higher than that without hydralazine [Hyd(−)] (Fig. 5A). The Hyd(+) group showed elevated temperatures, faster heating, and slower cooling than the Hyd(−) group, especially in the second and third therapy cycles. The CEM43 delivered by ultrasound in the presence [Hyd(+)] and absence [Hyd(−)] of hydralazine were respectively 170.8 ± 11.8 min and 137.7 ± 10.7 min (Fig. 5B). The difference in CEM43 between Hyd(−) and Hyd(+) groups was statistically significant (*p* < 0.05), which is equivalent to a 24.1% increase in thermal dose following hydralazine treatment. Notably, the tumor temperatures exceeded 45 °C for brief periods during the ultrasound therapy with or without hydralazine, but the cumulative thermal dose remained in the “moderate” hyperthermia regime^28^. In addition, the mice exhibited normal electrocardiogram, heartbeat, and breathing and no sign of skin burns following the ultrasound treatment with or without hydralazine.

### Areas of hemorrhage and vessel diameters increased in HCC following hydralazine and ultrasound treatment

**Figure 6:**
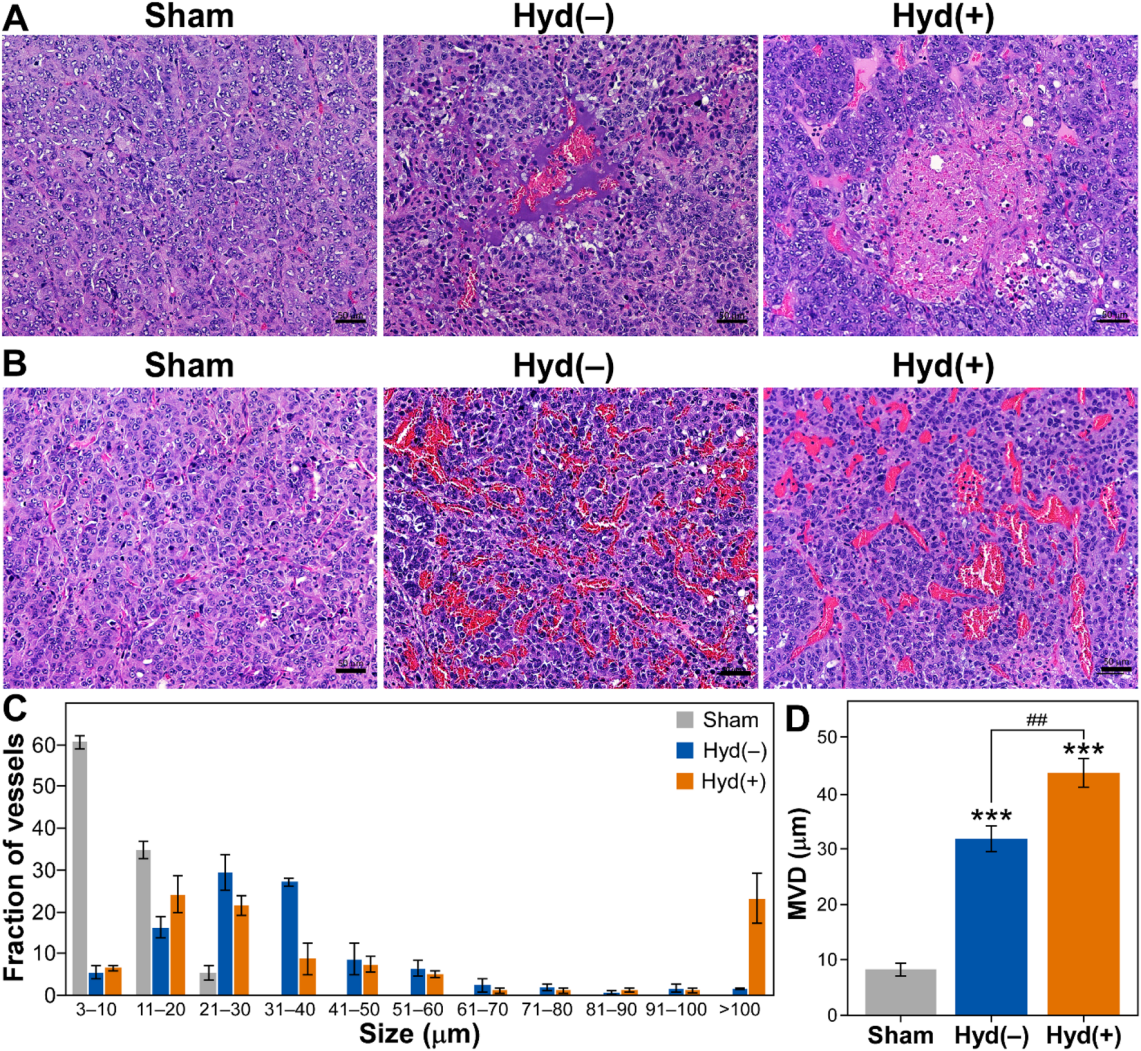
Histological examination of HCC. H&E-stained images of tumor samples showing hemorrhagic pools (**A**) and tumor vessels (**B**) in the sham-treated (Sham) or ultrasound-treated groups with [Hyd(+)] or without [Hyd(−)] hydralazine. Scale bar = 50 μm. In comparison to the sham group, there were obvious regions of hemorrhage following therapy, especially in tumors receiving hydralazine and ultrasound. (**C**) Histogram showing the distribution of tumor blood vessels. (**D**) Histogram showing the mean vessel diameter (MVD) of the tumor vessels. Difference in MVD between Sham vs. Hyd(−) (***p ≤ 0.001) and Sham vs. Hyd(+) (***p ≤ 0.001), and between Hyd(−) vs. Hyd(+) (^##^*p* ≤ 0.01) is statistically significant.

Histology showed a qualitative increase in size and number of tumor hemorrhage regions following the combined hydralazine and ultrasound treatment compared to ultrasound alone; such changes were not evident in the sham-treated tumors (Fig. 6A). Ultrasound hyperthermia with or without hydralazine resulted in the dilated tumor vessels, but these larger vessels were not present in the sham-treated tumors (Fig. 6B). Interestingly, the vessels over 100 μm in diameter were more prevalent in tumors treated with combined ultrasound and hydralazine [Hyd(+)] than in the tumors treated with ultrasound alone [Hyd(−)] (Fig. 6C). There was also a significant increase (*p* < 0.001) in the mean vessel diameter of the tumors treated either with ultrasound alone, [Hyd(−)] or ultrasound + hydralazine, [Hyd(+)]. The tumor vessel diameter increased significantly (*p* < 0.01) in [Hyd(+)] compared to [Hyd(−)]—mean vessel diameter (MVD) in [Hyd(+)] = 51.7 ± 4.5 μm; [Hyd(−)] = 32.4 ± 1.3 μm; sham-treated = 9.5 ± 0.6 μm (Fig. 6D).

## Discussion

We have previously shown in autochthonous HCC tumors developed in rats that ultrasound therapy in combination with intravascular microbubbles causes vascular disruption reducing in HCC perfusion^29,30^. Enhancement of vascular permeability by microbubble-assisted ultrasound has been shown in several types of cancer^31^. Ultrasound-induced antivascular effects have been attributed to enhanced microbubble oscillation by ultrasound stimulation generating higher shear, cavitation, and heating to the vascular endothelium^32,33^. Direct absorption of ultrasound by tissues also induces hyperthermia and has been a major factor in anti-cancer therapies^34^. Hyperthermia, alone or in combination with other therapeutic modalities such as radiation therapy, chemical drugs, or ablative ultrasound therapy has been shown to enhance HCC treatment^14,16,35^. Likewise, hyperthermia induced by ultrasound has been demonstrated in efficient drug delivery in tumors^36^. Several earlier studies have established high-intensity focused ultrasound (HIFU)-induced hyperthermia as a technology for cancer therapy in humans^37,38^, including HCC^39,40^. The safety and feasibility of the focused ultrasound-triggered drug delivery in clinical trials have been extensively discussed^41^. Contrast to HIFU, the current study utilizes a non-focused low-intensity ultrasound beam. The non-focused low-intensity ultrasound is non-ablative and treat lesions by increasing the temperature gradually to 45 °C or less. The non-focused nature of the beam allows treatment of larger lesions like HCCs which can be as large as 5 cm in diameter^42^. Because of gradual tissue heating and lower temperatures involved in hyperthermia, heat loss by blood flow becomes a critical factor in acquiring the desired temperature.

The heat loss problem is further exasperated if the hyperthermia therapy includes non-focused low-intensity ultrasound where the temperature increases are small and difficult to sustain for prolonged periods due to active blood flow. In this study, we postulated that hydralazine, a well-known peripheral vasodilator, selectively turns down the blood flow to the HCC tumors.

It has been reported earlier that hydralazine reduces tumor blood flow and enhances hyperthermia^24,43,44^. However, its use for enhancing therapeutic ultrasound in HCC tumors has not been shown. In the current study, CEUS imaging assessed the changes in blood flow induced by hydralazine-mediated hyperthermia. The results show reduced contrast enhancement following hydralazine administration indicating a marked reduction in intra-tumor blood flow. Hydralazine substantially attenuated time-intensity curves and reduced tumor perfusion measured by PE, PI, and AUC. Hydralazine administration decreased PE, PI, and AUC by more than 85%, and the reduction was statistically significant. The decrease in tumor blood flow by hydralazine is most likely related to the ‘steal’ phenomenon where hydralazine-induced dilation of the peripheral blood vessels siphons the blood away from the tumor due to the failure of the aberrant and immature tumor vasculature to dilate^45^.

Reduction of tumor blood flow by hydralazine offers a unique opportunity for hyperthermic therapies against HCC. The underlying hypothesis is that hydralazine—by reducing tumor blood flow—extends the thermal effects of ultrasound on HCC tumors. Consistent with the hypothesis, intravenous administration of hydralazine significantly enhanced the ultrasound-induced bioeffects in murine HCC tumors; namely, PE, PI, and AUC were markedly reduced compared to the hyperthermia performed with ultrasound alone. Likewise, the thermal dose delivered on mice tumors by ultrasound in the presence of hydralazine was significantly higher than that by ultrasound alone.

Broadly, hyperthermia is classified into mild, moderate, and ablative temperature regimes^28^. Mild hyperthermia entails heating to 40–41 °C for 24–72 h with CEM43 < 5 min. Moderate hyperthermia, on the other hand, involves tissue heating to 42–45 °C for 15–60 min with CEM43 of 15–240 min. Lastly, ablative hyperthermia involves heating to temperatures above 50 °C for 4–6 min with CEM43 > 512 min. The CEM43 of 137–170 min used in the current study is within the moderate hyperthermia regime. It is higher than that used for radiosensitization but lower than that used for ablative therapies^46^.

Ultrasound hyperthermia for cancer treatment has been induced by different sonication configurations including focused and planar ultrasound^47^. Various hyperthermia-generating devices with a broad range of capabilities involving mechanical and electronic scanning of the ultrasound beam and real-time temperature monitoring have been developed to achieve anti-tumor effect^33,48–50^. Amid the wide array of conventional hyperthermia approaches, this study delivers thermal dose through cyclic sonication where ultrasound was turned on and off for 2-min intervals. The cyclic sonication was used to prevent overheating of the tissue and the transducer. Since ultrasound absorption increases with frequency, the studies were performed at 2.8 MHz which is higher than that used clinically. The goal of our studies was to affect the microenvironment of the tumor. Histological analyses showed the basic architectural pattern and cellular structures of the tumor tissue were largely preserved and not destroyed by ablation. Several hemorrhagic pools were observed indicating the disruption of weak and loosely developed aberrant tumor microvasculature. Although the cumulative thermal dose remained in the moderate hyperthermia range and the mice well-tolerated the therapy, the temperature exceeded 45 °C for brief intervals during treatment. This was unavoidable due to the preset-fixed experimental treatment protocol of 2:2 min on:off cycles used in the study. A better control of temperature would be desirable in the future studies. Instead of using fixed on:off cycles, temperature of the tissue could be used to guide the treatment by turning off ultrasound when the temperature approaches 45 °C. Lower sonication intensities or longer duration of off-time could be deployed as other alternatives to maintain the temperature below 45 °C.

In this study, the temperatures were recorded at the surface of the tumors to maintain the integrity of the tumor vasculature. Although these measurements do not represent the whole tumor temperature, they show that the proposed ultrasound therapy effectively delivered adequate thermal dose in the tumors within 12 min compared to conventional hyperthermia which is performed typically over longer durations of 30–60 min^51^. The heating of the tumor, however, might not be uniform throughout the whole mass. Heterogeneities in tissue heating have been observed in clinical studies despite the uniform ultrasound fields^52^. The distribution of thermal dose in the tumor during the ultrasound therapy depends on several distinct but inter-related factors including regional variation in the heat deposition by the absorption of ultrasound along the path of propagation, and the differences in heat loss by conductivity and tumor blood flow. It has been demonstrated that the ultrasonic energy is attenuated in depth-dependent manner^53^; the thermal conductivity^49^ and blood flow^50^ at the tumor periphery are different than that in the tumor core. Depending on the relative magnitude of these factors, the tumor core and periphery can heat differently.

Hydralazine-induced augmentation of the ultrasound bioeffects was also observed in histological examinations of the H&E-stained tumor samples. The incorporation of hydralazine resulted in the formation of larger hemorrhagic pools and more dilated vessels. A marked increase in the number of larger tumor vessels ascertains the enhancement of thermal effects of ultrasound therapy. A similar progressive increase in the dilation of the central vein and sinusoids in rat livers by increase in exposure temperature has been observed previously^54^. Notably, the current data showed that the combined hydralazine and ultrasound hyperthermia resulted in a marked increase in larger vessels with a diameter larger than 100 μm. Such an increase could be of significance, as the minimum vessel size at which a substantial difference between blood and tissue temperature usually occurs has been reported to be between 100 and 400 μm^55^. The current hyperthermia therapy was intended to affect the tumor microvasculature without producing a substantial cell death directly. The histologic analyses demonstrated that the general cellular architecture and integrity are mostly conserved with an increase in several large hemorrhagic pools after ultrasound treatment even without hydralazine (US − Hyd). Such hemorrhagic pools were larger in the US + Hyd vs. US − Hyd-treated groups (Fig. 6). Tissue heating-induced increase in vascular damage, tumor hemorrhage, and necrosis has been reported earlier^56^. Therefore, hydralazine-induced augmentation of ultrasound hyperthermia also caused more vascular damage in tumors leading to increased blood pools and necrosis.

It would be relevant to note here that HCC vascularization is characterized by the presence of vessels encapsulating tumor clusters (VETC) pattern, which has been reported to be a strong prognostic markers in patients with HCC^57^. The HCC tumor vasculature in Hepa1-6 xenografts in mice also exhibit the similar VETC pattern^58^ suggesting its similarity with human HCC in terms of its blood flow and vascularity. However, as it is well recognized, the use of xenograft models does not completely emulate clinical models and more advanced clinical models would be required in the future studies to provide translation to human studies involving metastasis and angiogenesis.

Hyperthermia is known to affect cell physiology resulting in alterations in cell morphology and cytoskeletal rearrangements. Heat treatment incites the actin filaments disassembly, changes in microtubule morphology, and the collapse of intermediate filaments around the nucleus^59^. Collectively, the three major cytoskeletal filaments, namely, actin filaments, intermediate filaments, and microtubules, provide and maintain cell shape and structure and play key roles in tumor cell migration and metastasis^60^. Subsequently, hyperthermia results in cell membrane retraction, loss of microvilli, rounding up of cells, and blebbing of cell membrane^61^. Formation of such blebs on cell membrane has also been reported when cells were exposed to low-frequency^62^ and low-intensity^63^ ultrasound. Similarly, the formation of punctuated actin upon ultrasound stimulation has been reported earlier^64^. Additionally, hyperthermia can disrupt tight junctions between the endothelial cells and decrease the expression of tight junction protein zonula occluden-1^65^ occludin, claudin-5, and zonula occludens^66^. Mechanistically, ultrasound-induced opening up of the tight junctions have been attributed to mild hyperthermia caused by ultrasound stimulation^67^. These prior studies on the effect of hyperthermia on endothelial cell junction suggest that the observed increase in hemorrhagic pools and vasodilation in HCC are primarily due to the increased heating of the tumor induced by hydralazine. Mechanical effects including ultrasound-induced cavitation could play a role but are likely to be small due to the relatively low intensity of ultrasound. The current ultrasound hyperthermia is similar to microwaves, and other forms of heating such as radiant heat (visible and infrared), capacitive/inductive radiofrequency, and magnetic field heating of nanoparticles^47^. The current study can be developed further in the future for other tumor models besides HCC, such as melanomas and soft tissue sarcomas.

This study demonstrates the effect of hydralazine in a preclinical model but does not examine the hydralazine dosage-effect relationship. The choice of 5 mg/kg hydralazine dose was based on the results of a previous study in mice where a single intravenous injection of hydralazine significantly enhanced the local heat damage in a C3H mammary carcinoma in vivo^24^. Hydralazine doses of 1–10 mg/kg has been shown to exhibit significant anti-tumor effects in mouse fibrosarcoma^68^. The hydralazine dose of 5 mg/kg is also below the ‘standard dose’ of 50 mg/kg used for lowering blood pressure^69^; and the dosage of 7.5 mg/kg/day used clinically for the treatment of chronic hypertension in children and adolescents^70^. Although the dose of 5 mg/kg is within the realm of its use shown previously, the results should not be extrapolated to clinical hyperthermia without additional safety studies^71^. Rigorous pharmacokinetic studies are needed to determine the clinically acceptable safe hydralazine dosage of ultrasound hyperthermia. A substantial reduction in flow by hydralazine at 5 mg/kg suggests that there is potential to use lower doses of hydralazine for hyperthermia in the future.

It is known that hydralazine acts as a potent DNA methylation inhibitor^72^. Hydralazine binds directly to the catalytic region of DNA methyl transferase 1 (DNMT1) without incorporation into DNA^73^. Molecular modeling and molecular dynamics studies have also shown the binding of hydralazine to human DNMT1^74^. Recently, recognizing the significance of DNA methylation as a key driver of cancer^75^, several DNA methylation inhibitors^76^ including hydralazine^77–79^ have been studied in several cancer models. Thus, incorporation of hydralazine with ultrasound hyperthermia could also contribute to anticancer effects.

Lastly, hydralazine has been shown to decrease the total and low-density lipoprotein (LDL) cholesterol^80^. This raises the possibility of hydralazine-induced destabilization of the lipid-shelled contrast agents. However, hydralazine administration did not attenuate echointensity of the tissues adjacent to HCC tumors (Supplementary Fig. S1). Had the decrease in echogenicity been caused by hydralazine-lipid shell interaction, a reduced contrast enhancement would have been observed in both the tumors and the non-tumor tissues. Thus, the decrease in the echogenicity is less likely to be a manifestation of hydralazine-microbubbles interaction.

## Conclusions

In conclusion, this study demonstrated that the therapeutic efficacy of ultrasound hyperthermia can be significantly improved by the use of hydralazine for the treatment of highly vascular tumors such as HCC. Hydralazine, a widely used vasodilator, slowed down the HCC tumor blood flow and extended the thermal effects of ultrasound treatment in murine HCC tumors by modulating tumor vasculature and decreasing flow-mediated heat loss. The augmentation of ultrasound hyperthermia by hydralazine could have important clinical implications for HCC treatment in the future. However, clinical translation of the proposed method would require safety and optimum dose evaluation of ultrasound and hydralazine.

## Materials and methods

### Generation of HCC

Mouse hepatoma cells Hepa1-6 (ATCC CRL-1830; American Type Culture Collection, Manassas, VA, USA) were seeded and maintained in Dulbecco’s modified Eagle’s medium; supplemented with fetal bovine serum (Catalog # 26140095, Gibco, Gaithersburg, MD, USA) to a final concentration of 10%, 100 U/mL penicillin and 100 μg/mL streptomycin in a water-saturated atmosphere of 5% CO_2_ at 37 °C. In this xenograft model, Hepa1-6 cells (1 × 10^6^ cells per mouse), taken after 24 h of cell seeding, were implanted subcutaneously in the right flank of the mice.

### Animals

The animal studies were approved by the Institutional Animal Care and Use Committee (IACUC # 804998), University of Pennsylvania Office of Animal Welfare. Adult, male immunodeficient athymic nude mice (25–35 g; Charles River Laboratories, Wilmington, MA, USA) were accommodated in metabolic cages under controlled environmental conditions (25 °C and a 12 h light/dark cycle). Mice had free access to standard powdered and pelleted food and tap water ad libitum. All mice were studied during light cycles.

The growth of HCC tumors in mice was monitored routinely by ultrasound imaging (13–24 MHz; Vevo 2100 system—Fujifilm VisualSonics, Toronto, ON, Canada). Treatments were performed when the tumors grew to approximately 10 mm in diameter. Each mouse was moved to an acrylic box, and given general inhalational anesthesia with 1–2% isoflurane (USP Reference Standard, CAS # 26675-46-7, Halocarbon Laboratories, River Edge, NJ, USA) and 100–200 mL/min oxygen, following IACUC guidelines for mouse anesthesia. The animal was then placed on a heated table in a supine position; anesthesia was maintained via a nosecone.

### Treatment with hydralazine alone

The effects of intravenous hydralazine (5 mg/kg; CAS # 304-20-1, Sigma, St. Louis, MO, USA) on HCC blood flow were studied in two groups (n = 5) of mice. In the current study, one group received intravenous hydralazine and the other sham-treated group an identical volume of normal saline. Before and following treatment, CEUS images of each tumor were acquired (13–24 MHz; Vevo 2100 system—Fujifilm VisualSonics, Toronto, ON, Canada) after the intravenous injection (via a tail vein) of 10 μL of contrast-enhancing perflutren lipid microspheres (Definity, Lantheus Medical Imaging, North Billerica, MA, USA). CEUS images were acquired 5 min after hydralazine injection or saline injections. Imaging presets (gain = 18 dB; high sensitivity; 100% power; transmit frequency 21 MHz; high line density) and time-gain-compensation were optimized and kept fixed for all CEUS imaging. Grayscale and nonlinear contrast-enhanced (NLC) images were acquired before and after contrast injection. The enhancement of images by microbubble inflow and outflow was analyzed for the qualitative and quantitative assessment of HCC vasculature. Grayscale images were used to manually outline a region of interest (ROI) delineating the tumor margins. The ROI from the grayscale image was superimposed on the NLC image to assess echo-intensity values over time to obtain the time-intensity curves based on the bolus perfusion model using software (Vevo CQ) on the scanner. Peak enhancement (PE), perfusion index (PI), and area under the curve (AUC) were calculated from the time-intensity curves. PE is the maximum signal intensity reached during the transit of the contrast bolus; AUC is the time integral of the gamma variate function and corresponds to blood volume; PI corresponds to blood flow and is the ratio of AUC to the average time for the blood to pass through the region of interest. The values for each parameter were averaged over each group of mice and the mean [± standard error of the mean (SEM)] values were compared between the two groups.

### Combined ultrasound and hydralazine treatments

The effects of combined ultrasound and hydralazine treatments were studied on HCC blood flow in three groups of mice (n = 5) using a similar method described earlier^81^. Briefly, after the intravenous injection of 5 mg/kg hydralazine, an initial group was treated with non-focused plain wave ultrasound beam (frequency = 2.8 MHz; spatial-average-temporal-peak intensity (I_SATP_) = 1.6 W/cm^2^; peak pressure = 0.23 MPa; continuous mode; three 2:2 min on and off cycles). Sonication was done with a 15-mm-diameter ultrasound transducer with the near field at 10.2 mm. The ultrasound transducer was placed on the tumor surface aligning the center of the tumor with the center of the ultrasound beam corresponding to the heating field. Tumors were located at the depth of approximately 4.2 ± 0.3 mm from the surface of the transducer. Many studies^41,82^ have used ultrasound frequency on the order of 1 MHz for cancer treatments, and several other studies^47,83^ have used higher frequency ultrasound (1–5 MHz) as well. As ultrasound absorption increases near-linearly with frequency, the 2.8 MHz frequency was used in the current study to maximize tissue heating. The 2:2 min on:off cycles of sonication were used to minimize any direct heating of the transducer surface, and prevent excessive thermal dose delivery.

In a second group, hyperthermia was performed without hydralazine and a third control group received a sham therapy where the ultrasound hyperthermia machine was not turned on and hydralazine was also not administered. Prior to and following treatment, CEUS images of each tumor were acquired as described above. The values for each parameter were averaged over each group of mice and the mean (± SEM) values were compared between the two groups.

### Tumor temperature and thermal dose measurement

Tumor temperature for each ultrasound treatment (with or without hydralazine) was measured using the approach described earlier^81^. Briefly, a fine-wire 0.08-mm-diameter thermocouple (Omega Engineering Inc., Stamford, CT, USA) was placed under the exterior surface of the live mouse tumor, and the temperature was recorded every second during the therapy. The thermocouple was aligned perpendicular to the ultrasound probe and placed about 1 to 2 mm outside the 15-mm-wide ultrasound beam. Thermal dose (TD) was computed by the formula^84,85^:

$$\mathrm{TD}= {\int }_{0}^{t}{R}^{(T-43)}dt,$$

where t = time (min); R = 4 for T < 43 °C; and R = 2 for temperature T ≥ 43 °C. Thermal dose at temperature ≥ 43 °C was expressed in terms of cumulative equivalent minutes at 43 °C (CEM43)^47^.

### Histochemical staining and analyses

After each study, the mouse was euthanized by cervical dislocation. Necropsy was performed right after euthanasia, and the tumor was harvested for histologic examination. Tumors were preserved in 10% phosphate-buffered formalin for 48–72 h before being transferred to 50% ethanol, embedded in paraffin, and finally processed for histological examination with hematoxylin and eosin (H&E) stain. Digital microscopic images of H&E sections were acquired (ZEISS Axio Imager 2, Carl Zeiss Microscopy GmbH, Jena, Germany); and the diameters of the tumor neovasculature were measured and analyzed using software ImageJ^86^. Briefly, the diameter of the vessels was measured and averaged from the images chosen from five randomly-selected locations of the tumors of each animal.

### Statistical analyses

Statistical analyses were performed using SPSS software (IBM SPSS Statistics, IBM Corp., Armonk, NY, USA). The normality of each experimental group was evaluated by Shapiro–Wilk test. The *p*-value > 0.05 was used to accept the normal distribution of the data. To test the statistical difference between the means (± SEM) of the independent groups, one-way analysis of variance (ANOVA) was performed followed by Tukey’s test as a post hoc test; *p* ≤ 0.001 (***), *p* ≤ 0.01 (**) and *p* ≤ 0.05 (*) were considered significant and *p* > 0.05 was considered not significant (ns).

### Image editing

All the figures were generated by compiling and editing the original images in Microsoft PowerPoint 2016 software.

### Ethical approval

The authors confirm that all methods were carried out in accordance with relevant guidelines and regulations. The authors also confirm that the study was carried out in compliance with the ARRIVE guidelines (http://www.nc3rs.org.uk/page.asp?id=1357).

## Supplementary Information

Supplementary Figure S1.
