# Supplementary material for: Hydralazine augmented ultrasound hyperthermia for the treatment of hepatocellular carcinoma
Source: Sci Rep. 2021 Jul 30;11:15553. doi: 10.1038/s41598-021-94323-0 (PMC8324788; doi:10.1038/s41598-021-94323-0)
Supplement: Supplementary file 1 — Supplementary Figure S1. [file 41598_2021_94323_MOESM1_ESM.docx]

**Hydralazine Augmented Ultrasound Hyperthermia for the Treatment of Hepatocellular Carcinoma**

Mrigendra B. Karmacharya^§^, Laith R. Sultan^§^, Stephen J. Hunt, and Chandra M. Sehgal.

^§^These authors have contributed equally in this study.

Department of Radiology, Perelman School of Medicine, University of Pennsylvania, 3620 Hamilton Walk, Philadelphia, PA 19104, USA.

**Corresponding Author**: Chandra M. Sehgal, Ultrasound Research Laboratory, Department of Radiology, Perelman School of Medicine, University of Pennsylvania, 3620 Hamilton Walk, Philadelphia, PA 19104, USA. Tel: 215-573-4963; Fax: 215-898-6115; Email: [Chandra.Sehgal@pennmedicine.upenn.edu](mailto:Chandra.Sehgal@pennmedicine.upenn.edu)

**Supplementary Figure**

**
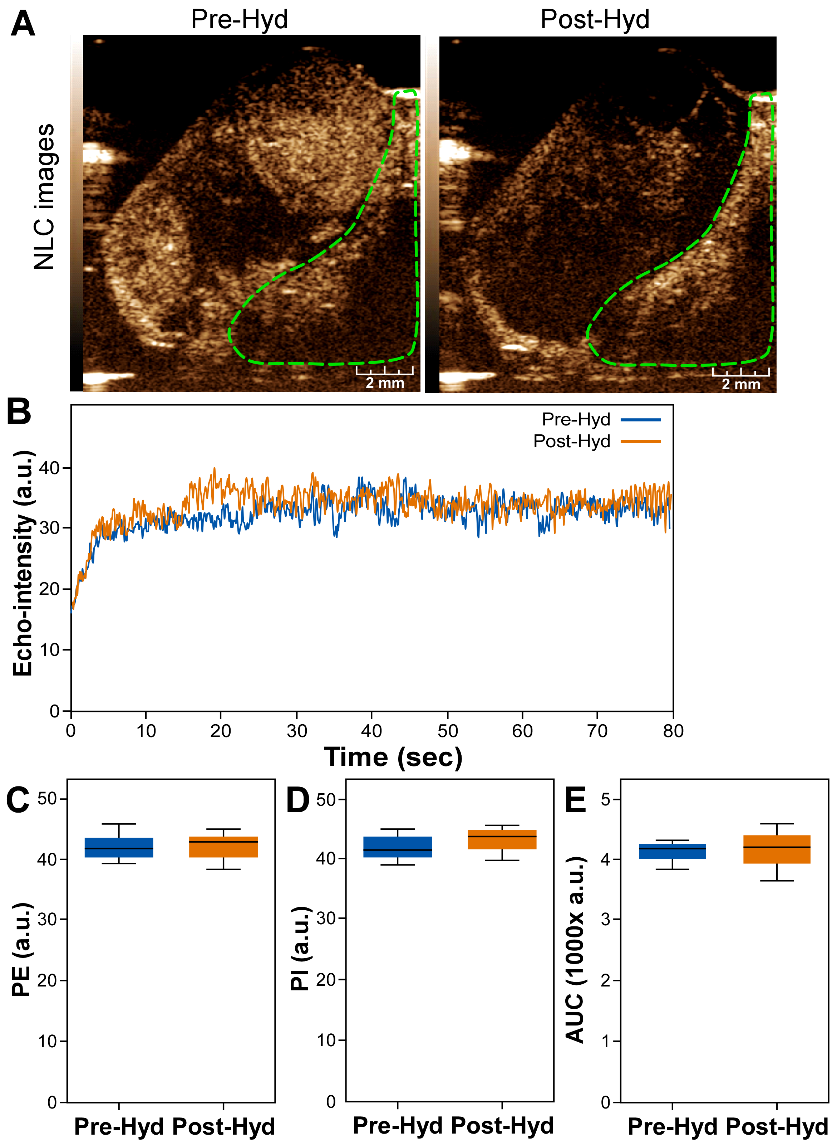
**

**Supplementary Figure S1:** **Effects of hydralazine on blood flow in the adjacent tissue of HCC tumors. (A)** Contrast-enhanced non-linear contrast (NLC) images of the tumor acquired before (Pre-Hyd) and after (Post-Hyd) intravenous injections of 5 mg/kg hydralazine; the margins of the tumor defined by the green dotted lines were outlined on the grayscale images (not shown) and copied to the NLC image acquired simultaneously. Scale bar = 2 mm. The time**-**intensity curves (**B**) displaying echo-intensity (arbitrary units, a.u.), plotted over time before (Pre-Hyd) and after (Post-Hyd) hydralazine treatment. Note that hydralazine did not decrease echogenicity in the adjacent tissue of HCC tumors. Boxplots (**C**), (**D**), and (**E**) illustrate the five-number summary, the minimum, first quartile, median, third quartile, and maximum value of peak enhancement (PE), perfusion index (PI), and area under the curve (AUC) respectively. All data points for PE, PI, and AUC for Pre-Hyd and Post-Hyd groups were normally distributed by the Shapiro-Wilk test (*p*-value > 0.05). Hydralazine treatment did not show any reductions in the CEUS vascular imaging parameters in the adjacent tissue of HCC tumors.
